# Comparing saliva and urine samples for measuring breast milk intake with the ^2^H oxide dose-to-mother technique among children 2–4 months old

**DOI:** 10.1017/S0007114519002642

**Published:** 2019-12-16

**Authors:** Eric Matsiko, Paul J. M. Hulshof, Laura van der Velde, Marlou-Floor Kenkhuis, Lisine Tuyisenge, Alida Melse-Boonstra

**Affiliations:** 1 Division of Human Nutrition and Health, Wageningen University and Research, PO Box 17, 6700 AA Wageningen, The Netherlands; 2 Department of Human Nutrition and Dietetics, University of Rwanda, PO Box 3286 Kigali, Rwanda; 3 Department of Pediatrics, University Teaching Hospital of Kigali, PO Box 655 Kigali, Rwanda

**Keywords:** Breast milk intake, ^2^H oxide dose-to-mother technique, Doubly labelled water, Saliva samples: Urine samples, Maternal energy expenditure

## Abstract

Saliva and urine are the two main body fluids sampled when breast milk intake is measured with the ^2^H oxide dose-to-mother technique. However, these two body fluids may generate different estimates of breast milk intake due to differences in isotope enrichment. Therefore, we aimed to assess how the estimated amount of breast milk intake differs when based on saliva and urine samples and to explore whether the total energy expenditure of the mothers is related to breast milk output. We used a convenience sample of thirteen pairs of mothers and babies aged 2–4 months, who were exclusively breastfed and apparently healthy. To assess breast milk intake, we administered doubly labelled water to the mothers and collected saliva samples from them, while simultaneously collecting both saliva and urine from their babies over a 14-d period. Isotope ratio MS was used to analyse the samples for ^2^H and ^18^O enrichments. Mean breast milk intake based on saliva samples was significantly higher than that based on urine samples (854·5 *v*. 812·8 g/d, *P* = 0·029). This can be attributed to slightly higher isotope enrichments in saliva and to a poorer model fit for urine samples as indicated by a higher square root of the mean square error (14·6 *v*. 10·4 mg/kg, *P* = 0·001). Maternal energy expenditure was not correlated with breast milk output. Our study suggests that saliva sampling generates slightly higher estimates of breast milk intake and is more precise as compared with urine and that maternal energy expenditure does not influence breast milk output.

The WHO recommends to exclusively breastfeed children during the first 6 months of life^(1)^. In this period, breast milk should preferably be the sole source of nourishment for the child’s growth and development^(2)^. Exclusive breast-feeding imparts the child with health benefits and reduces the risk of childhood morbidity and mortality^(3,4,5)^. To determine the adequacy of breast milk quantity and nutrient intake and to link breast-feeding patterns to children’s growth and development, accurate quantification of breast milk intake is essential.

Breast milk intake used to be quantified by test weighing methods, for which the child’s weight is taken before and after each breastfeed, and the difference between these two weights amounts to breast milk ingested by the child^(6,7)^. However, this method is time-consuming and disturbs breast-feeding routine. Another disadvantage is the inability to assess if a child is exclusively breastfed because the test weighing method does not capture water intake from other sources^(8)^. In surveys, the prevalence of exclusive breast-feeding is usually based on maternal recalls, which are often associated with recall bias and socially desirable responses that lead to over-estimation of the true prevalence^(9)^.

To overcome these challenges, a more objective technique called the ‘^2^H oxide dose-to-mother technique’ involving the use of stable isotopes was developed^(8,10)^. The ^2^H oxide dose-to-mother technique, first described by Coward in 1980^(11)^, was found to give comparable estimates of breast milk intake to the test weighing method^(10)^. The major advantage of the stable isotope technique is that daily breast milk intake is estimated over a 14-d period without interfering with the breast-feeding routine or being too much of a burden to mothers^(8)^. Additionally, with this technique, a child can either be classified as exclusively breastfed or not. The technique is based on the ^2^H enrichment of the body fluids of both the mother and the child. For the easiness of collection, either saliva or urine is usually preferred as body fluid^(12,13)^. However, in studies with labelled water, the level of isotope enrichment has been found to differ between types of body fluids, with saliva samples having a slightly higher isotopic enrichment than urine samples^(12–15)^. Therefore, the use of either of these types of body fluids is likely to result in different outcomes^(16)^. To this end, Rieken *et al*. assessed the comparability of saliva and urine samples to quantify energy expenditure and body composition and they concluded that both types of body fluids give comparable estimates^(16)^. However, Jankowski *et al*. and Schierbeek *et al*. found that, as opposed to urine, saliva provides a more accurate estimate of the intended outcome^(12,13)^.

Although both saliva and urine have been sampled in studies to measure breast milk intake with the ^2^H oxide dose-to-mother technique^(6,17–19)^, it is not known how the type of body fluid affects the estimate for breast milk intake. Therefore, we aimed to quantify and compare estimates of breast milk intake with the ^2^H oxide dose-to-mother technique when based on either saliva or urine samples among 2- to 4-month-old children. In addition, since we expected large differences in physical activity patterns between mothers, we simultaneously measured maternal energy expenditure by using doubly labelled water and explored how this was related to breast milk output.

## Methods

### Study site and participants

The study was conducted in a rural area of Muhanga District, Southern Province, Rwanda, and in the town of Wageningen, the Netherlands. In the Netherlands, we collected samples in the homes of the mothers during the autumn of 2015, and in Rwanda during the rainy season. The Rwandan National Ethics Committee and the Medical Ethics Committee of Wageningen University and Research approved the present study. We followed the ethical guidelines as laid down in the declaration of Helsinki and its amendments. In accordance, the study objective and procedures were explained to parents both verbally and in writing before they gave their written consent.

**Fig. 1.**
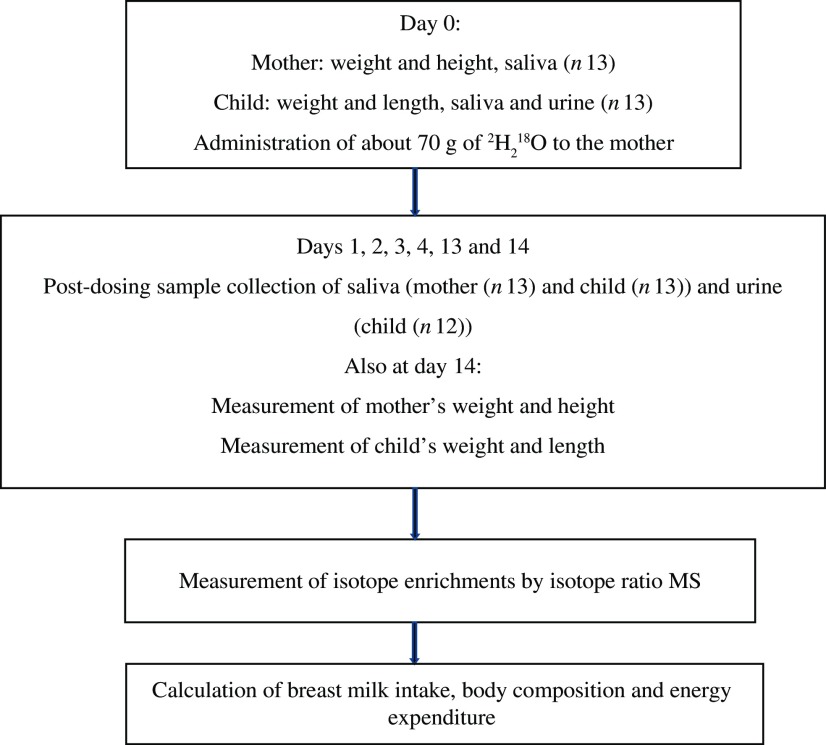
Study flow diagram: summary of the main steps of the study.

A convenience sample of thirteen mother–child pairs (five Dutch and eight Rwandans) took part in the study. The Rwandan participants were recruited through the Rutobwe health centre, and Dutch participants were recruited at the local swimming pool during baby swimming sessions and at child consultation clinics. The recruited mothers were exclusively breast-feeding (reported by mother), apparently healthy, and were willing to stay in the study areas for the next 2 weeks. The children were aged 2–4 months, full-term, singleton and were apparently healthy. Fig. 1 summarises the flow of the study.

### Preparation and administration of doubly labelled water doses

The doubly labelled water mixture was prepared several days before the start of the dosing. Before aliquoting individual doses of approximately 70 g, doubly labelled water was filtered using Whatman puradisc FP 30/0·2 syringe filters (GE Healthcare Europe GmbH) and dispensed into a 250 ml polyethylene bottle. The prepared doses were stored overnight in a refrigerator until administered to the mothers. Mothers received a mixture of 1·8 g 10 % enriched H_2_^18^O (Centre for Molecular Research Ltd) and 0·3 g 99 % enriched D_2_O (Cambridge Isotope Laboratories, Inc.) per kg body water. It was assumed that the body weight of females comprised 50 % of body water^(20)^. In human studies for breast milk output and body composition, the ^2^H consumed by study participants enriches the body water to a maximum of 0·1 %, which is a safe level and far lower than 15 % at which harmful effects can occur^(21,22)^.

Each mother drank an accurately weighted dose of approximately 70 g from the polyethylene bottle using a straw. Immediately after drinking the dose, we rinsed the bottle with approximately 100 ml drinking water and the mother drank this water rinse as well using the same straw. The time when the mother drank the dose was recorded on data collection sheets.

### Data and sample collection

MSc students from Wageningen University and Research collected samples from both study sites. These students were trained in sample collection and study procedures by one investigator until they complied with the sample collection procedure. During the study implementation, the same protocol was used for both sites. We used the UNICEF electronic scale and length or height boards to measure weight and length (child) or height (mother), respectively. These anthropometric measures were collected in duplicates from mothers and children at baseline (day 0) and at day 14 of the study (Fig. 1). Weight was recorded to the nearest 0·1 kg and height or length to the nearest 0·1 cm.

After baseline anthropometric measures and before the mother drank the dose (day 0), we collected 2 ml of saliva from both mother and child and 5 ml of urine only from the child. Subsequent urine and saliva samples were collected on 1–4, 13 and 14 d post-dosing (Fig. 1).

To collect maternal saliva samples, the mother held a sterile cotton ball in her mouth until saturated with saliva. The saliva-saturated cotton ball was then transferred into a 20 ml syringe, and saliva was squeezed into a 2 ml cryogenic vial. Cotton swabs were used to collect saliva samples from the child. We kept the cotton swabs moving in the child’s mouth for about 2 min. The saliva-sodden cotton swabs were then placed into a syringe to express saliva into a 2 ml cryogenic vial. This process was repeated two to three times to collect enough saliva from the child (1–2 ml).

We collected urine samples from children only. After cleaning genital parts, the child wore a diaper fitted with a cotton inlay pad. Once a child urinated, we removed the cotton inlay pad and placed it into a plastic bag. After cutting a corner tip from the plastic bag, we manually squeezed out urine into a cup. Of this, 5 ml of urine was immediately transferred into a cryogenic vial. We recorded the sampling time of all samples on data collection sheets.

#### Storage of samples

The sealing caps of the cryogenic vials were wrapped with parafilm for tight closure. On the day of collection, the cryogenic vials were transported in a cooler box to the local laboratory at the University of Rwanda and stored in a freezer at −20°C until they were transported frozen to the laboratory of the Division of Human Nutrition and Health, Wageningen University, The Netherlands.

### Analysis of samples for the isotope enrichment

All samples were flame-sealed in 25 µl pre-calibrated pipettes (Vitrex Medical A/S). Isotopic enrichment of the saliva samples, urine samples and diluted doses was analysed at the Center for Isotope Research, Groningen, The Netherlands, as described elsewhere^(23)^. Briefly, the urine samples in the capillaries were subjected to a micro-distillation procedure to obtain pure distilled water. Next, a volume of 0·12 µl of distilled water was injected using an auto-sampler (CTC PAL, CTC Analytics) through a heated septum into a high-temperature pyrolysis unit consisting of a glassy carbon reactor with a temperature >1300°C (Hekatech). The reaction products of the pyrolysis process (H_2_ and CO_2_ gasses) were led by a continuous flow of He gas on a GC, where the gasses were separated and led into a continuous flow isotope ratio MS (IRMS) system (Isoprime, GV Instruments). Each sample was injected six times for δ^2^H analysis followed by three more injections for δ^18^O analysis. Ratios (R) of C^18^O/C^16^O and ^3^H/^2^H relative to reference water (VSMOW, Vienna Standard Mean Ocean Water) were expressed in δ units of ‰ after correction for memory effects. The relative difference between sample isotope ratio and the isotope ratio of the international standard was expressed as delta units using this formula: δ^18^O or δ^2^H (‰) = 1000 × (Rs–R_VSMOW_)/R_VSMOW_). Enrichments expressed as delta units were converted into parts per million excess^(24,25)^. ^2^H and ^18^O dilution spaces were calculated using the intercept method.

The reference water (biomedical enriched waters gravimetrically prepared from VSMOW) was analysed for quality control and showed analytical variations of <0·5 % for both isotopes, and accuracy defined as deviation from the certified values was <1 % for δ^2^H and <0·3 % for δ^18^O.

### Calculations of breast milk intake and water intake from other sources

A multipoint protocol was used for concurrently estimating breast milk intake of the children and the energy expenditure of the mothers. We calculated breast milk intake according to Haisma *et al*.^(26)^ The calculations were based on fitting the ^2^H enrichment data to a model for water turnover in the mother and child. We used solver function in Microsoft excel to fit data of enrichment to the following equations:
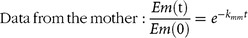
where *E*_*m(t)*_ is the ^2^H enrichment in the mother’s body water at time *t*, in mg/kg; *t* is the time since the dose was taken; *E*_*m(0)*_ is the ^2^H enrichment in the mother’s body water at time zero, mg/kg; *k*_*mm*_ is the fractional water turnover in the mother (kg/d).

Data from the child: 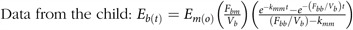



where *E*_*b(t)*_ is the ^2^H enrichment in the baby’s body water at time *t*, in mg/kg; *t* is the time since the dose was taken by the mother, in d; *E*_*m(0)*_ is the ^2^H enrichment in the mother’s body water at time zero (mg/kg); *F*_*bm*_ is the transfer of water from the mother to the child via breast milk (kg/d); *V*_*b*_ is the baby’s total ^2^H distribution space (kg). *V*_*b*_ was assumed to change linearly with weight over study period, *V*_*b*_ = 0·84 W^0·82^; *k*_mm_ is the fractional water turnover in the mother (kg/d); *F*_*bb*_ is the total water loss in the child (kg/d).

The amount of breast milk intake was calculated from the water flow from the mother to the child assuming that 87·1 % of breast milk is water^(27)^. Therefore, *F*_*bm*_/0·871 gives breast milk intake (g/d).

### Calculation of child and maternal body composition

The calculated components of the body composition were total body water (TBW), fat mass and fat-free mass. TBW was calculated as the average of the ^2^H dilution space divided by 1·041 and ^18^O dilution space divided by 1·01 to account for non-aqueous isotope exchange^(28)^. We calculated the fat-free mass (FFM, kg) of the mothers as TBW (kg)/0·732, assuming that 73·2 % of FFM is water^(29)^. The difference between body weight and FFM gave fat mass. For the children, the FFM was calculated as TBW (kg)/*h*_FFM_ with *h*_FFM_ being hydration constant of the FFM, which was assumed to be 0·80 for boys and 0·79 for girls^(30)^.

### Calculation of maternal total energy expenditure

Isotope elimination rates (k_O_ and k_D_) were calculated as the gradient of the plot of the natural logarithm of the enrichment in body water *v*. time since the dose was taken. The rate of carbon dioxide production was calculated using the following formula: rCO_2_ (litres per d) = 0·455 × TBW (litres) × (1·007 k_O_–1·041 k_D_)^(24)^ and total energy expenditure using the modified Weir equation^(31)^. Total energy expenditure (kJ/d) = rCO_2_ (litres per d) × (1·1 + 3·90/RQ) × 4·184, where RQ is respiratory quotient and was assumed to be 0·85.

### Statistical analysis

We compared the mean difference between breast milk intake based on saliva and urine with paired and independent *t* tests. The square root of the mean squared error (MSE) was used to evaluate the goodness of the modelled data fit, which reflects the difference between the measured and model-predicted ^2^H enrichments in the mother and child. The *P* value was set at 0·05 for each statistical test of significance. To assess the patterns of the differences between breast milk intake based on saliva and urine, the differences between breast milk intake based on saliva and urine were plotted against the means of two intakes using a Bland-Altman pairwise comparison.

## Results

Table 1 presents the characteristics of the study participants. The mean age and mean body weight of Rwandan children were slightly higher than those of Dutch children. The body fat mass percentage of Dutch children (25·7 (sd 2·3) %) was slightly higher than that of Rwandan children (23·7 (sd 5·1) %). The age of mothers ranged from 21 to 38 years with a mean of 30 years. Dutch mothers tended to have a slightly higher mean body weight and fat mass, and they were taller on average. Nonetheless, the BMI of Dutch mothers (23·2 (sd 4·7) kg/m^2^) was comparable with that of Rwandan mothers (23·4 (sd 2·6) kg/m^2^). In addition, the mothers had similar FFM (41 (sd 3·4) kg).

**Table 1.** Characteristics of the study participants (Mean values and standard deviations; numbers of participants)

	Dutch			Rwandan			All		
Characteristics	Mean		sd	Mean		sd	Mean		sd
Number of mother–child pairs		5			8			13	
Child									
Sex ratio (male:female)		1:4			5:3			6:7	
Age (months)	3·4		1·0	3·7		0·6	3·6		0·7
Weight at day 0 (kg)	6·0		0·5	6·8		1·7	6·5		1·4
Weight at day 14 (kg)	6·4		0·7	6·6		0·9	6·8		1·3
Weight gain (g/d)	20·8		17·3	19·6		18·5	20·2		17
Length (cm)	61·7		1·7	60·5		2·5	61·0		2·3
BMI-for-age *z*-scores	0·14		1·3	0·99		1·8	0·66		1·6
Body water (kg)	3·7		0·2	3·9		0·6	3·9		0·5
Fat-free mass (kg)	4·7		0·3	5·0		0·7	4·9		0·6
Fat mass (kg)	1·6		0·3	1·5		0·4	1·6		0·3
Fat mass (%)	25·7		2·3	23·7		5·1	24·5		5·1
Mother									
Age (years)	30·8		0·8	30·5		6·1	30·6		4·6
Body weight at day 0 (kg)	63·8		13·2	60·2		5·3	61·5		8·8
Height (cm)	165·3		3·3	160·9		7·2	162·6		2·2
BMI (kg/m^2^)	23·1		4·7	23·4		2·6	23·2		3·4
Total body water (kg)	30·2		2·4	30·0		2·7	30·0		2·5
Fat-free mass (kg)	41·0		3·4	41·0		3·7	40·9		3·4
Fat mass (kg)	22·5		13	19·3		3·6	20·5		8·1
Fat mass (%)	33·2		10·6	31·9		4·4	32·4		7

Table 2 shows the quality parameters of the kinetic data based on analysis with GC-pyrolysis-isotope ratio MS. Table 3 compares the isotope kinetic results between saliva and urine samples. The ^2^H enrichment tended to be higher in saliva samples than in urine samples at each time point. Additionally, the overall mean of ^2^H enrichment and AUC were slightly higher for saliva samples (89·3 and 1505 parts per million) than that for urine samples (87·4 and 1461 parts per million). However, enrichments were only statistically different for the AUC (*P* = 0·009).

**Table 2.** Kinetic parameters for analysis of doubly labelled water

	*R* ^2^			Abundance at 14 d		Isotope elimination rate: k_O/_k_D_
Participant	^2^H	^18^O	Dilution space: ^2^H/^18^O	^2^H	^18^O	
D1	0·998	0·998	1·037	56·9	29·9	1·2
D2	0·999	0·999	1·012	81·6	21·8	1·3
D3	0·999	0·999	1·005	71·5	22·8	1·2
D4	0·994	0·997	0·997	69·3	22·8	1·2
D5	0·999	0·999	1·006	70·5	22·6	1·2
RW1	0·999	0·999	1·046	41·5	16·8	1·3
RW2	0·998	0·999	1·069	51·8	23·6	1·3
RW3	0·999	0·999	1·047	48·3	22·6	1·3
RW4	0·999	0·999	1·061	48·5	25·3	1·2
RW5	0·999	0·998	1·030	60·6	26·7	1·3
RW6	0·999	0·999	1·014	47·5	22·7	1·2
RW7	0·999	0·994	1·018	42·3	17·0	1·3
RW8	0·998	0·999	1·043	48·9	23·0	1·3
Range	0·994–0·999		0·997–1·069	41·5–81·6	16·8–29·9	1·2–1·3

*R* ^2^, coefficient of the determination regression line; ^18^O, oxygen-18; k_O_, oxygen-18 elimination rate; k_D_, ^2^H elimination rate; D, Dutch; RW, Rwandan.

**Table 3.** Kinetic results based on saliva and urine body fluids (Mean values and standard deviations)

Outcome	Saliva				Urine				Saliva		Urine		
	Dutch		Rwandan		Dutch		Rwandan		All		All		
	Mean	sd	Mean	sd	Mean	sd	Mean	sd	Mean	sd	Mean	sd	*P* *
^2^H enrichment (ppm) *T*_1*S*_ = *23·6, T*_1*U*_ = 23·9	53·2	11·0	43·6	11·50	50·0	9·9	39·7	10·6	47·4	11·7	43·8	11·1	0·049
^2^H enrichment (ppm) *T*_2*S*_ = 48·2, *T*_2*U*_ = 48·8	102·3	10·6	78·0	19·24	98·3	17·3	75·9	21·4	86·8	21·1	84·0	22·2	0·090
^2^H enrichment (ppm) *T*_3*S*_ = 71·8, *T*_3*U*_ = 72·6	131·8	16·6	95·3	24·9	130·1	17·9	88·8	22·3	109·9	28·1	105·3	28·9	0·031
^2^H enrichment (ppm) *T*_4*S*_ = 96·0, *T*_4*U*_ = 96·4	151·0	14·8	113·9	19·4	154·2	15·8	108·4	17·3	127·4	25·3	125·1	28·0	0·371
^2^H enrichment (ppm) *T*_13*S*_ = 317·5, *T*_13*U*_ = 317·9	115·1	7·5	74·5	10·2	113·0	11·0	71·9	11·8	88·1	21·2	85·6	23·0	0·054
^2^H enrichment (ppm) *T*_14*S*_ = 342·0, *T*_14*U*_ = 342·8	106·8	8·3	67·0	9·0	106·4	8·2	66·1	9·6	80·3	20·5	79·5	21·6	0·313
Mean ^2^H enrichment (ppm)	110·0	10·5	78·6	14·7	108·6	11·6	76·8	14·8	89·3	20·0	87·4	20·5	0·106
AUC (ppm)	1994	226·2	1283·8	202·4	1924·4	253·3	1230·0	202·1	1504·1	382·0	1461·5	400·4	0·009
Square root MSE (mg/kg)	12·8	11·0	9·1	2·9	17·2	8·1	13·3	5·0	10·4	6·4	14·6	6·1	0·001
Breast milk output (g/d)	760·2	65·6	901·6	261·2	748·7	77·1	844·8	221·2	854·5	222·3	812·8	187·1	0·029
Non-milk oral water intake (g/d)	−11·7	16·2	34·2	115·8	−7·5	20·5	70·5	129·7	18·9	95·5	44·5	110·9	0·022
Daily energy intake (kJ/d)†									2502·0	652·7	2380·7	548·1	0·029

T, time (h) of sample collection after dosing; S, saliva; U, urine; ppm, parts per million; MSE, mean squared error, which is the differences between the measured and model-predicted ^2^H enrichment in the mother and child.

* *P* value between saliva and urine estimates of each outcome for all participants.

† Energy intake is estimated based energy density of 2·93 kJ/g according to Dewey *et al*.^(32)^.

The square root of MSE in data from saliva samples (10·4 (sd 6·4) g/d) was significantly lower compared with that in data from urine samples (14·6 (sd 6·1) g/d), *P* = 0·001). Furthermore, average breast milk intake based on saliva samples was higher (854·5 (sd 222·3) g/d) than that based on urine samples (812·8 (sd 187·1) g/d), *P* = 0·029. Moreover, saliva samples resulted in significant lower estimated non-breast milk water intake. At the individual level, for ten out of twelve mother–child pairs, saliva provided higher breast milk intake estimates compared with urine and only one child was classified as not exclusively breastfed based on non-breast milk water intake estimated from either saliva or urine (data not presented). The calculated energy intake based on both types of samples showed that saliva-based breast milk intake provided significantly higher energy intake than urine-based breast milk intake (2502 *v*. 2377 kJ/d, *P* = 0·029) (Table 3).

**Fig. 2.**
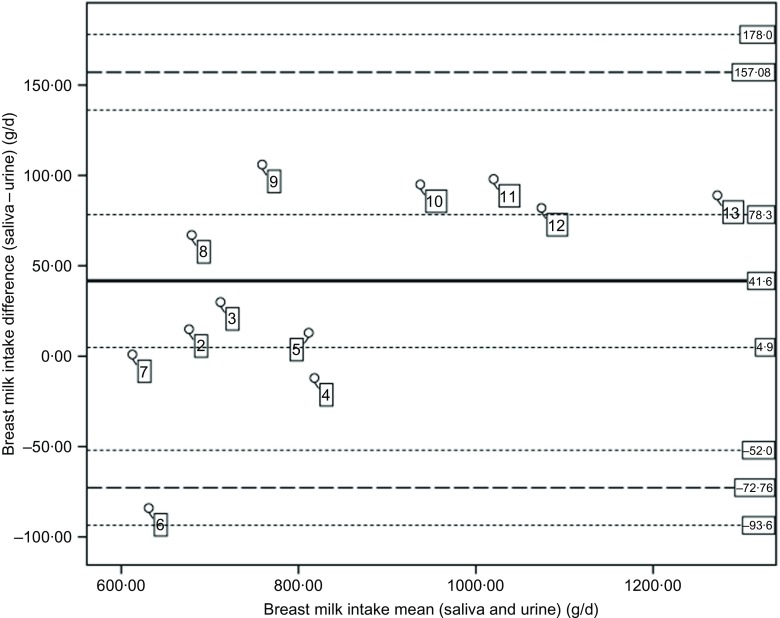
Bland and Altman plot of the differences of breast milk intakes (saliva – urine) plotted against mean intakes ((saliva + urine)/2). The central plain line is the mean difference in breast milk intakes from saliva and urine (41·6 g/d). The lower and upper thick dashed lines indicate the mean difference plus two standard deviations (mean, 2sd = −72·76, 157·08). The thin dotted lines indicate the CI for the mean (4·9, 78·3), lower limit (−93·6, −52) and the CI for the upper limit (136·2, 178·0). The numbers in the figure represent the participants per country (2–5 for Dutch and 6–12 for Rwandans).

Mean breast milk output based on saliva (760 (sd 65·6) g/d) was higher but not significantly different from the mean intake based on urine samples (749 (sd 77·1) g/d) among Dutch participants (*P* = 0·27) (Table 3). In contrast, saliva samples provided significantly higher mean breast milk intake (901 (sd 261·2) g/d) compared with urine samples (844 (sd 221·2) g/d) among Rwandan participants (*P* = 0·045). For both types of body fluid, the mean breast milk intake of Dutch children was lower, but not significantly different from that of Rwandan children (*P* = 0·70 for saliva and *P* = 0·42 for urine) (Table 3) and Lin’s correlation coefficient test showed a high concordance between saliva and urine samples (*r* 0·94). Fig. 2 shows individual differences between breast milk intakes based on saliva and urine.

**Fig. 3.**
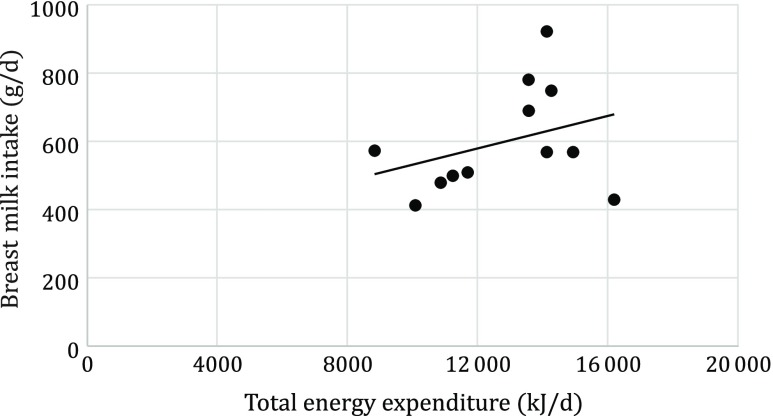
Correlation between maternal energy expenditure and breast milk intake (*r* 0·33; *P* = 0·284).

Mean total daily energy expenditure was significantly higher in Rwandan mothers than in Dutch mothers (13 480 (sd 1966) *v*. 10 695 (sd 2414) kJ/d, *P* = 0·043). Maternal total daily energy expenditure did not significantly correlate with breast milk intake, neither for the total sample (*r* 0·33, *P* = 0·28) (Fig. 3) nor for the Dutch and Rwandan group separately (Dutch: *r* 0·05,*P* = 0·88; Rwandan: *r* 0·27, *P* = 0·50). Breast milk intake did not also correlate with mother’s age (*r* 0·006, *P* = 0·98) and mother’s body fat (*r* 0·29, *P* = 0·32), but it did correlate with child’s BMI-for-age (*r* 0·80, *P* = 0·001), child’s FFM (*r* 0·62, *P* 0·032), and with the mother’s BMI (*r* 0·60, *P* = 0·036).

## Discussion

The present study showed that saliva samples resulted in approximately 5 % higher estimates of breast milk intake than urine samples and that maternal energy expenditure did not correlate with breast milk intake. There are two main reasons that can explain the observed difference between saliva and urine as media for a breast milk intake assessment. First, it may be due to a higher level of ^2^H enrichment in saliva compared with urine and secondarily to a poorer fit of the enrichment data based on urine samples.

In our study, saliva samples were slightly more enriched in ^2^H than urine samples. This finding is in accordance with earlier observations by Schoeller *et al*.^(15)^ and Schierbeek *et al*.^(12)^ who found a similar difference in enrichment patterns between saliva and urine. This observed difference in ^2^H enrichment may be related to fractionation patterns, that is, the relative abundance of ^2^H oxide isotopes in body fluids^(13)^. Since breast milk intake is quantified based on ^2^H enrichment^(8)^, the different levels of ^2^H enrichment between saliva and urine samples are therefore likely to influence the magnitude of breast milk intake estimates. Accordingly, Rickien *et al*. attributed a lower estimate of energy expenditure to a slightly lower isotopic enrichment in urine compared with saliva^(16)^.

To quantify breast milk intake, the ^2^H enrichment data are fitted to model for water turnover in mother and child^(8)^. With the model, the square root of MSE is calculated to assess the fit of the modelled data. The smaller the square root of MSE, the more precise the data^(33)^. In our study, the square root of MSE is significantly smaller for saliva samples than for urine samples indicating a poorer fit when urine samples are used. This is probably caused by, on the one hand, the longer time taken by tracers to equilibrate in the contents of the bladder, and on the other hand, the time lag between initial urine production in the kidney and sample collection, particularly in children who are still incontinent at younger age^(14,34)^. Therefore, the urine-based data resulted in larger random errors as opposed to saliva-based data. These larger random errors together with a relatively small sample size (twelve participants) may have contributed to less reliable measurements based on urine samples. Consequently, this may have resulted in a small but statistically significant difference in estimated breast milk intake between the two types of sample media. However, based on a cutoff of 86·6 g/d for classifying exclusivity of breast-feeding^(35)^, a difference of 5 % between two sample media seems to be less important and Lin’s correlation coefficient test shows that both types of samples were highly correlated.

Although the mean breast milk intake based on saliva and urine samples differs, each of these mean intakes exceeds the pooled mean breast milk intake of 820 g/d reported for exclusively breastfed 3- to 4-month-old children^(36)^. Likewise, based on estimated mean energy intakes of 2502 kJ/d (saliva-based breast milk) and 2376 kJ/d (urine-based breast milk), children in the present study would meet, for example, the energy need of 2384 and 2184 kJ/d for a 3-month-old boy or girl, respectively^(37)^. In addition, for individual children in the present study, a similar proportion of children (50 %) would meet their daily energy needs by consuming breast milk as estimated based on either saliva or urine. Thus, a mean difference of not more than 5 % between saliva and urine samples does not affect estimates of energy adequacy; however, Fig. 2 shows that the variability in differences between breast milk intake based on the two types of samples is higher at lower intake.

From a practical perspective, we experienced that collecting urine samples was challenging due to the uncontrollable time of the release of urine by the child, sometimes resulting in a long waiting time and frequently disturbing the child for checking the wetness of the cotton inlay pad. This waiting time was sometimes lengthened when a child defecated, which required us to change the diaper and inlay pad. For these reasons, urine collection was more cumbersome contrary to saliva. Taken together, saliva, therefore, seems to be more suitable as a medium for studies using the ^2^H oxide dose-to-mother technique than urine.

Breast milk intake did not statistically differ between Dutch and Rwandan children. Brown *et al*. also reported that breast milk intake in developing countries does not differ from intake in developed countries^(38)^. Mean breast milk intakes estimated in the present study are approximatively within the intake range of 744–925 g/d reported in other studies using the ^2^H oxide dose-to-mother technique^(10,17–19,26,31,39)^. In addition, despite a small sample size, the breast milk intake in our study also compares well with approximately 700–800 g/d reported by Dewey *et al*. who used the test weighing method^(40)^.

Since we used doubly labelled water in the present study, we measured maternal body energy expenditure in addition to breast milk intake. The energy expenditure of Rwandese mothers was significantly higher than that of Dutch mothers. This finding agrees well with what Singh *et al*. found in Gambian and English lactating mothers^(41)^. The rural livelihood conditions for the Rwandan study mothers, dominated by farming activities, are the basis of the observed difference in energy expenditure. Rural residents are generally more active than urban residents^(42)^, and adult Africans generally participate more in vigorous-intensity physical activity than Europeans^(43)^. In addition, a study conducted in the Gambia showed that the energy cost of physical activity among mothers during the season of highest farming activities was up to 2·5 times higher than that of affluent non-farming mothers^(41)^. These factors explain the difference in energy expenditure between Dutch and Rwandan mothers. Nevertheless, energy expenditure did not correlate with the quantity of breast milk in either setting. Therefore, it seems that energy expenditure in lactating mothers does not affect breast milk output in the present study, as has also been reported earlier from studies on maternal exercise and lactation performance^(44,45)^.

The strength of the present study is that it used an objective technique to measure breast milk intake and energy expenditure. In addition, the quality of the enrichment data in the present study was acceptable. This is shown by the accuracy, precision and other details of the kinetic data based on analysis with GC-pyrolysis-isotope ratio MS (Table 2), which were within an acceptable range and comparable to other studies^(24)^. Additionally, the study compares the findings between two different settings. However, the small sample size is the major limitation of our study, and therefore, results are not representative of the respective source populations. In addition, possible differences in conditions during collection (such as humidity and temperature) between the study sites could have resulted in different fractionation of the isotopes and therefore differential retention of the isotopes in the body, which consequently affected breast milk intake estimates. However, we estimate the impact to be limited: in the Netherlands, the samples were collected in autumn (from 28 September to 1 December 2015) in the homes of the mothers. The room temperature and humidity in the homes were not recorded, but can be expected to be between 16 and 22°C. In Rwanda, the samples were collected from 15 April to 5 May 2015. This corresponded to the rainy season with an average temperature of 18°C.

To conclude, saliva samples resulted in higher estimates of breast milk intake than urine samples. The difference between the two types of body fluid may mainly be attributed to differences in ^2^H enrichment and to larger random errors in urine data indicating a poorer fit. The collection of urine samples is more cumbersome compared with saliva. Therefore, both from a methodological and from a practical perspective, saliva sampling is preferable over urine sampling in studies measuring the amount of breast milk intake with the ^2^H dose-to-mother technique. Energy expenditure in lactating mothers does not affect breast milk output.
